# Advanced 3D Cell Culture Technologies and Formats

**DOI:** 10.3390/bioengineering12060606

**Published:** 2025-06-03

**Authors:** Cornelia Kasper, Dominik Egger

**Affiliations:** 1 Department of Biotechnology and Food Science, BOKU University, 1190 Vienna, Austria; cornelia.kasper@boku.ac.at; 2 Institute of Cell Biology and Biophysics, Leibniz University Hannover, 30419 Hannover, Germany

The Special Issue “Advanced 3D Cell Culture Technologies and Formats” presents a collection of original research and review articles that explore recent innovations in three-dimensional (3D) cell culture systems aimed at enhancing the physiological relevance and therapeutic utility of cells and cell-derived products and assays. Conventional two-dimensional cultivation is known to impose artificial constraints on cell performance, often leading to altered phenotypes, impaired functionality, and reduced translational value. In contrast, 3D culture technologies offer more biomimetic environments that support improved cell–cell and cell–matrix interactions, thereby better preserving cellular characteristics and function. The studies featured in this Special Issue address key challenges in the field, including the development of functional biomaterials, the engineering of scalable bioreactor systems, and the application of 3D models for disease modeling and drug development. Collectively, the contributions demonstrate the potential of advanced 3D culture formats to improve the quality and applicability of in vitro systems for both fundamental research and therapeutic development.

The highly recognized review “In vitro 3D modeling of neurodegenerative diseases” by Louit et al. provides a comprehensive overview on recent developments and studies applying novel technologies and formats in the field of neurodegenerative diseases with a focus on Alzheimer’s, Parkinson’s, and Huntington’s diseases, as well as amyotrophic lateral sclerosis [1]. The authors introduce the benefits and limitations of 3D in vitro models and comprehensively describe different formats and methods for generating models for disease modeling. Different advanced and beyond-state-of-the-art technologies are presented and discussed. Tang-Schomer et al. tackle the critical lack of in vitro models for ependymoma, a malignant pediatric brain tumor, by developing a 3D culture system that mimics the tumor microenvironment [2]. By integrating VEGF, extracellular matrix components, and endothelial cells, the authors successfully recapitulate key features of patient-derived tumor vasculature and preserve patient-specific transcriptomic profiles. Their model offers a promising platform for studying tumor biology and advancing personalized therapeutic strategies for ependymoma. Type I diabetes is a disease based on defect or lost pancreatic cells resulting in insufficient insulin production, leading to dysregulated blood-glucose levels in patients. Amin et al. present the application of functionalized hydrogels for embedding pancreatic cells with the aim of improving insulin production [3]. The authors apply alginate as a biomaterial with RGD peptide functionalization for the embedding of primary mouse beta cells in spheroids and they study the effect of biomaterial stiffness on the overall aim of enhancing insulin secretion.

Jeske et al. present a novel concept applying a self-designed and optimized concept for a specialized bioreactor for dynamic mesenchymal stem cell (MSC) aggregate cultivation [4]. The authors aim to improve the yield and potency of MSC-derived EV and show that dynamic wave-motion improved the aggregation of the MSC and promoted EV production. The paper also provides supplemental material on specific details of the optimization steps involved. One of the major limitations in cell therapy application still remains the need for generating a sufficient amount of biological active cells. Baudequin et al. developed and optimized a beads-based system for the scalable expansion of MSCs [5]. In this study, the authors focus on the chosen functionalization of the used biomaterial for the cultivation of MSC.

A critical challenge in the field of liver microphysiological systems (MPSs) is addressed by the study of Lim et al.: the need for reproducibility and robustness across varied experimental conditions to support broader adoption in drug development [6]. By systematically evaluating the PhysioMimix LC12 platform with different hepatocyte sources and non-parenchymal cell combinations, the authors demonstrate that primary human hepatocytes deliver superior metabolic function, while induced pluripotent stem cell (iPSC)-derived hepatocytes and complex co-cultures show limited performance. Their findings offer a validated framework for assessing MPS robustness under realistic use cases, paving the way for a more informed implementation in preclinical toxicology. Sieni et al. explore how microenvironmental inhomogeneities influence the electric field distribution and cellular response during electroporation—a technique increasingly used in biomedical applications [7]. Using finite element modeling, they demonstrate that both cell aggregation and extracellular matrix composition, particularly the presence of collagen, significantly affect the transmembrane potential. Their findings, validated through experiments with the human epithelial cell line (HCC195) in hyaluronic acid-based scaffolds, highlight the importance of tissue architecture in optimizing electroporation efficiency, with potential implications for targeted therapies and tissue engineering.

Together, the contributions to this Special Issue illustrate the breadth and depth of current advancements in 3D cell culture technologies. From innovative biomaterials and dynamic culture systems to disease-specific and scalable therapeutic models, these studies underscore the exceptional role of 3D formats in bridging the gap between in vitro experimentation and clinical application. As the field continues to evolve, such interdisciplinary approaches will be essential to fully harness the therapeutic potential of cells and their products.
